# Peripheral extremity gangrene following infant oral mutilation (Ebinyo): a case report from Northern Uganda

**DOI:** 10.4314/ahs.v25i2.11

**Published:** 2025-06

**Authors:** Mary Ann Etling, Venice Omona, Adrona A Nampogo Kyozira, Robert Natumanya

**Affiliations:** 1 Indiana University School of Medicine, Indianapolis, Indiana, USA; 2 Richard M. Fairbanks School of Public Health, Indianapolis, Indiana, USA; 3 Department of Pediatrics, St. Mary's Hospital Lacor, Gulu, Uganda

**Keywords:** Infant oral mutilation, ebinyo, gangrene

## Abstract

**Background:**

“Ebinyo” is a form of infant oral mutilation (IOM) practiced by some traditional healers in parts of sub-Saharan Africa, and often results in severe health complications. We present a novel case of ebinyo in a child from northern Uganda which resulted in sepsis and disseminated intravascular coagulation (DIC) with peripheral extremity gangrene.

**Case Presentation:**

A 3-year-old male presented with two weeks of epistaxis, oralbleeding, and cough to a large referral hospital in northern Uganda. At home, the child had undergone ebinyo, performed by a traditional healer, and was receiving treatment for malaria. On arrival, the child presented with fever, jaundice, and malaise. Labs revealed pancytopenia and an elevated D-dimer, diagnosed as DIC. On day four, physical exam revealed demarcated darkening of the fourth digit on the right foot and third and fourth digits on the left foot, diagnosed as dry gangrene. A doppler ultrasound revealed lower extremity arterial insufficiency. On day seven, the patient started improving with vancomycin. On day eleven, the patient was discharged.

**Conclusion:**

This case describes an uncommon sequela of sepsis and DIC with peripheral extremity gangrene after undergoing ebinyo. Prompt identification of ebinyo is critical for diagnosis and management of its complications.

## Background

“Ebinyo” is a form of Infant Oral Mutilation (IOM) performed by traditional healers, where the unerupted teeth, tonsils, and other oral structures are removed. The traditional practice is believed to prevent or treat common symptoms such as fever, vomiting, or diarrhea in young children. The term “ebinyo” is loosely translated to “false teeth” in the Bantu language. It is believed that the practice started in Nilotic Sudan in 1932, but it is practiced across sub-Saharan Africa with cases reported in Uganda, Chad, Sudan, Ethiopia, Somalia, the Democratic Republic of Congo, Kenya, Tanzania, Rwanda, and Burundi^1^. In northern Uganda, hospitalized admissions after ebinyo had a 21% mortality rate^2^. Here we describe a 3-year-old child who was brought to St. Mary's Hospital Lacor in northern Uganda after undergoing ebinyo in the local community.

## Case Presentation

A 3-year-old male of African origin was brought to Lacor Hospital in Gulu, Uganda by his mother following epistaxis and continuous oral bleeding. The mother reported that the child had developed fever, cough, and loose stool two weeks ago, so she took the child to a traditional healer who recommended ebinyo, a local practice where the tooth buds, tonsils, and/or uvula are removed. In this case, the tonsils had been removed bilaterally. The mother had also taken the child to the local health center where he received treatment for malaria. The child had no known medical conditions.

On arrival to Lacor Hospital, the child appeared ill with pallor and jaundice. The child was febrile at 37.9° C, and his blood pressure was 80/46 mmHg. The heartrate was 154 beats/minute and the child was saturating at 97% on room air. No bleeding was noted in the oral cavity, but the child had melena. On auscultation, the child had normal bilateral air entry, but episodes of grunting and coarse crackles were noted.

Investigations included CBC which revealed pancytopenia with a leukocyte count of 4.0×109 cells/L, hemoglobin of 7.8 g/dl, and a platelet count of 24×109 cells/L. The child was given a blood transfusion, D10 infusion, and initiated on ampicillin and gentamycin.

The child was placed on 1-liter supplemental oxygen. Over the next few days, the child remained sick-looking and febrile, up to 39.6° C. On exam, the oral cavity and teeth were soiled with blood, but no palpable lymph nodes or petechiae were noted. The mother reported that the child was not tolerating feeds well and had vomited. The child was dyspneic and drowsy with increased tone in the upper limbs with occasional tremor. Patient was started on ceftriaxone and metronidazole, and a nasogastric tube was placed for feeds. CBC revealed persistent thrombocytopenia with a platelet count of 18×109 cells/L. An mRDT was positive, so the child was started on artesunate.

**Figure 1:**
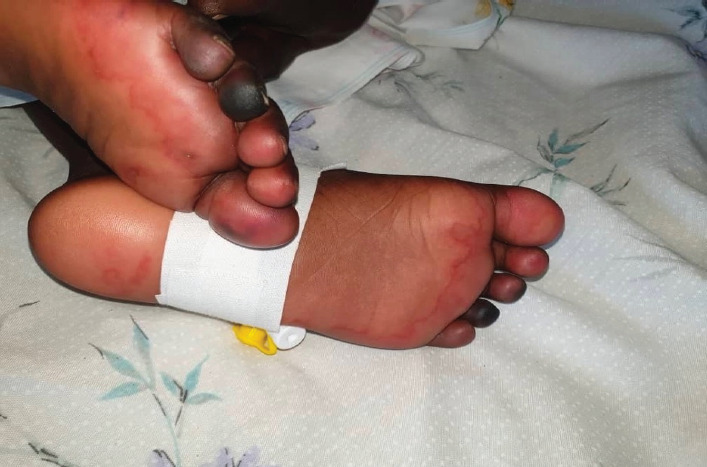
Image of the left and right feet of the patient on Day 4

**Figure 2:**
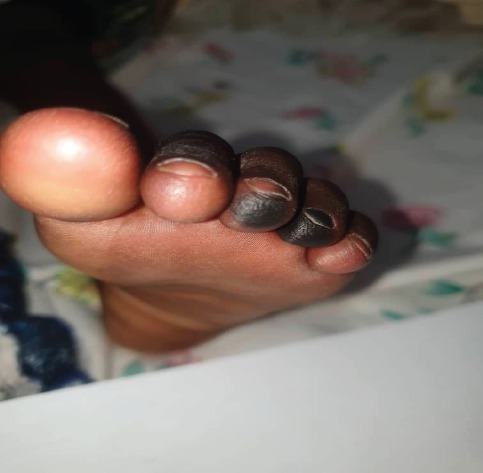
Image of the left foot of the patient on Day 4

**Figure 3:**
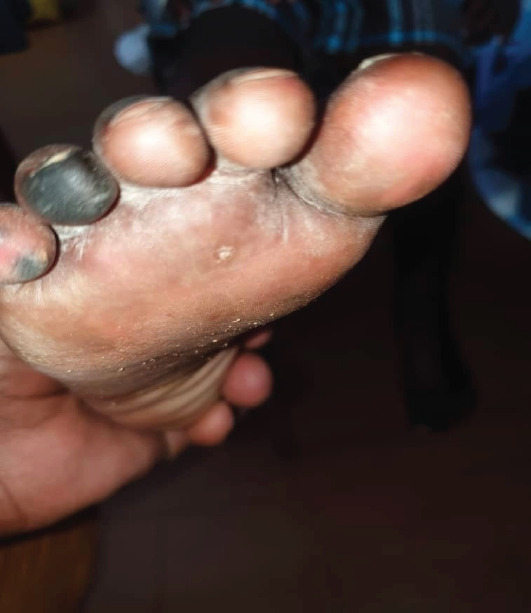
Image of the right foot of the patient on Day 4

**Figure 4:**
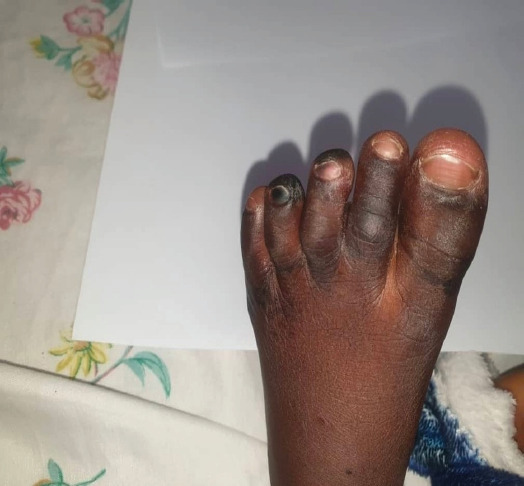
Image of the left foot of the patient on Day 4

**Figure 5:**
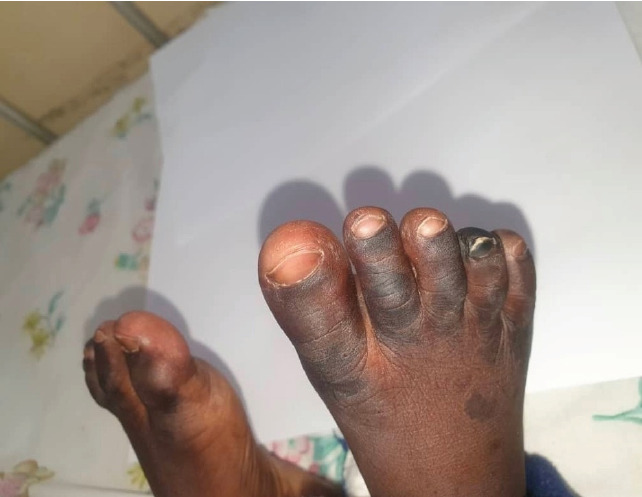
Image of the left and right feet of the patient on Day 4

On day four, the child presented with demarcated darkening of the fourth digit on the right foot and third and fourth digits on the left foot concerning for dry gangrene (see Figures 1-5), but the upper limbs were spared. Doppler ultrasound returned showing end arterial insufficiency in the toes bilaterally, but all other arteries of the lower limbs were of normal caliber and revealed no thrombosis. The dorsalis pedis and posterior pulse at the malleolus were palpable. CBC revealed slight leukocytosis (10.05×109 cells/L) with lymphocytic predominance (40.9%), worsening anemia (hemoglobin 8.3 dl/mg), and improved thrombocytopenia (62×109 cells/L). The D-dimer was elevated at 4635 ng/ml. The child was started on vancomycin, oral paracetamol, and oral morphine. Further investigations were ordered, including a repeat CBC and blood culture.

On day seven, the mother reported that the child had improved and was able to feed regularly. The child was afebrile (37.2° C) and asleep but arousable, saturating on 95% on room air. For the following three days, the child continued on antibiotics and feeding.

The blood culture returned on day eleven, which revealed no bacterial growth. On discharge, the darkening of the digits was gradually resolving and only involving the distal tip of the toes.

## Discussion

Ebinyo is the traditional practice of removing the unerupted teeth, tonsils, and other oral structures^1^. Reports of ebinyo have come from across Uganda, but incidence in the northern region has increased as much as three times in recent years^3^-^4^. The patient presented at St. Mary's Hospital Lacor, a 483-bed capacity hospital approximately six kilometers from Gulu, Uganda. Lacor Hospital serves as a referral site for health centers and clinics throughout the northern region. The staff at Lacor Hospital conducted a thorough social history identifying ebinyo, and immediately recognized the high risk for infection. The patient was stabilized with supplemental oxygen, a blood transfusion, intravenous fluids, antipyretics, and antibiotics.

The pathogenesis of sepsis-induced disseminated intravascular coagulation (DIC) leading to ischemia and gangrene is likely based on clinical findings, lab results, and imaging. The sepsis was likely caused in this case from an infection that originated from the oral mutilation, which likely used unsterilized instruments. The pathogenesis of sepsis causing DIC is well-understood. As the organism becomes bloodborne, various procoagulants may be released inappropriately, inducing widespread clot formation of small and medium vessels and depleting the body of platelets and clotting factors^5^.

When not corrected, the occlusion of vessels may lead to ischemia, necrosis, and endorgan damage. In this patient, the occlusion of lower limb vessels from sepsis induced DIC led to the gangrenous necrosis, noted on the toes bilaterally. Lab results and imaging supported a diagnosis of DIC with a markedly elevated D-dimer (4635 ng/ml), thrombocytopenia (62×109 cells/L), and doppler ultrasound returned showing end arterial insufficiency bilaterally.

A case of sepsis and DIC with peripheral extremity dry gangrene after undergoing ebinyo has never been reported in the literature. The majority of studies describe the impact on dental and oral health, citing hypoplasia, midline shift, dental displacement, missed or unerupted canine, and other permanent impact on occlusive status, negatively affecting oral health-related quality of life^6^-^9^. One case report describes noma, or orofacial gangrenous infection, secondary to ebinyo in western Uganda^10^.

Another case report from Kenya discusses a child who developed sepsis and DIC after ebinyo, leading to a fatality, but physical exam did not reveal gangrene^11^. A study based at Lacor Hospital found that one-fourth of hospitalized children in northern Uganda had died due to ebinyo, secondary to sepsis or anemia. The study described 209 admissions due to ebinyo in a four-week period and described ebinyo as having the third highest case fatality rate following meningitis and malaria^12^.

The primary limitation in this setting is diagnostic testing and antibiotic resistance. While there is a full-functioning laboratory and access to various diagnostic tools at Lacor Hospital, it can take a few days to receive results, which can delay medical decisionmaking. In this case, the staff ordered a blood panel and doppler ultrasound on day four but did not receive results until day six. Another limitation in this setting is antibiotic resistance. While it is difficult to determine in this case since a blood culture was not collected earlier and the later blood culture was negative, the patient did not clinically improve on ampicillin, gentamycin, ceftriaxone, or metronidazole, which cover a wide range of gram-positive and gram-negative organisms. However, when the patient was placed on vancomycin and artesunate for concurrent malaria, the patient improved.

Infant oral mutilation is still relatively widespread in sub-Saharan Africa, so interventions are necessary in order to reduce its use in the community. In rural Uganda, a nutrition and hygiene education intervention on oral health behavior was implemented and resulted in a significant reduction in the use of ebinyo with an incidence of 8.9% compared to 24.7% in the control group^13^. In this setting, traditional healers continue to have a substantial role in health decisions made by caregivers. As a result, it is imperative that interventions that increase awareness of the harm of IOM be utilized to educate traditional healers, dental practitioners, healthcare professionals, and community members^14^.

## Conclusion

Infant oral mutilation continues to impact the health of children in sub-Saharan Africa, specifically in northern Uganda. In rare cases, this traditional practice may result in a sequela of sepsis, DIC, and peripheral extremity gangrene. This case exemplifies how a thorough social history and prompt identification of ebinyo is critical for diagnosis and management of its complications.

## Data Availability

All data generated or analyzed during this study are included in this published article.

## Abbreviations

CBC: Complete blood count
DIC: disseminated intravascular coagulation
INR: International normalized ratio
IOM: Infant oral mutilation
mRDT: Malaria rapid diagnostic test
